# Interspecific Hybridization Is an Important Driving Force for Origin and Diversification of Asian Cultivated Rice *Oryza sativa* L.

**DOI:** 10.3389/fpls.2022.932737

**Published:** 2022-06-30

**Authors:** Jiawu Zhou, Ying Yang, Yonggang Lv, Qiuhong Pu, Jing Li, Yu Zhang, Xianneng Deng, Min Wang, Jie Wang, Dayun Tao

**Affiliations:** ^1^Yunnan Key Laboratory for Rice Genetic Improvement, Food Crops Research Institute, Yunnan Academy of Agricultural Sciences, Kunming, China; ^2^Institute of Plant Resources, Yunnan University, Kunming, China

**Keywords:** rice, origin, evolution, interspecific hybridization, introgression, diversity, adaptation

## Abstract

As one of the most important crops, Asian cultivated rice has evolved into a complex group including several subgroups adapting various eco-climate-systems around the globe. Here, we pictured a comprehensive view of its original domestication, divergences, and the origin of different subgroups by integrating agriculture, archeology, genetics, nuclear, and cytoplasm genome results. Then, it was highlighted that interspecific hybridization-introgression has played important role in improving the genetic diversity and adaptation of *Oryza sativa* during its evolution process. Natural hybridization-introgression led to the origin of *indica, aus*, and *basmatic* subgroups, which adapted to changing cultivated environments, and produced feral weedy rice coexisting and competing with cultivars under production management. Artificial interspecific hybridization-introgression gained several breakthroughs in rice breeding, such as developing three-line hybrid rice, new rice for Africa (NERICA), and some important pest and disease resistance genes in rice genetic improvement, contributing to the stable increase of rice production to meet the expanding human population. We proposed a series to exploit the virtues of hybridization-introgression in the genetic improvement of Asian cultivated rice. But some key issues such as reproductive barriers especially hybrid sterility should be investigated further, which are conducive to gene exchange between cultivated rice and its relatives, and even is beneficial to exploiting interspecific hybrid vigor. New technologies help introduce favorable genes from distant wild species to Asian cultivated rice, such as transgenic and genome editing systems. Rising introgression lines in a wider range with multi-donor benefits allele mining, understanding genetic network of rice growth and development, yield formation, and environmental adaptation. Then, integration of new tools and interspecific hybridization can be a future direction to develop more usable breeding populations which can make Asian cultivated rice more resilient to the changing climate and world.

## Introduction

The process of plant domestication often leads to genetic bottlenecks. Hybridization supplied chances to increase plant genetic diversity and adaptation ability with its expansion progress. Hybridization has been an important force in generating angiosperm species diversity (Soltis and Soltis, 2009). At least 25% of plant species, especially the youngest species, are involved in hybridization and potential introgression with other species (Mallet, 2005). It occurs when distinct populations, subspecies, or species come into contact, hybridize, and the gene pools are merged (Mallet, 2007). This often leads to the formation of a hybrid zone or a hybrid swarm (Nolte and Tautz, 2009). Hybridization can result in rapid genomic changes, which may lead to beneficial new phenotypes (Baack and Rieseberg, 2007). Carolus Linnaeus proposed a radical evolutionary hypothesis that new species could arise via hybridization (Larson, 1968). Interspecific hybridization occurred or is carried out in many plants (Kopecký et al., 2022), such as tomato (Lippman et al., 2007), cotton (Zhang et al., 2014), soybean (Wang et al., 2019), rice (Purugganan, 2019), grape (Foria et al., 2022), tobacco (Mino et al., 2022), and oak (Fu et al., 2022), served as an important mechanism for their genetic diversification, environment adaptation, and range expansion, as well as improvement of economical traits. Older crops, such as rice, also have longer time to diversify under cultivation and thus adapt to local environments as their geographic ranges widened (Milla and Osborne, 2021).

Without domesticated crops, very few members of human societies would survive if they had only a field of wild grain and herbs and their wits to sustain them (Doebley et al., 2006); thus, the continuous progress of domesticated crops improvement is the vital foundation for human survival, progress, and prosperity. Asia cultivated rice, *Oryza sativa*, is one of the most important crops to feed the world for thousands of years. It is believed to be domesticated from the wild relative, *Oryza rufipogon*, in East Asia, then evolved and expanded to form different climatic ecotypes over the world. Regarding the domestication of the Asian cultivated rice, one hypothesis is independent domestication of *indica* and *japonica*, the prototype of *O. sativa* evolved primarily along the foothills of the Himalayas in South Asia and its associated mountain ranges in mainland Southeast Asia and Southwest China, and the genetic differentiation developed parallel to the ecologic diversification process (Chang, 1976). Another hypothesis is that the domestication processes of Asian rice occurred only once, and the *japonica* originated from domesticated *indica* (Oka, 1988). The cloning and haplotype analysis of some domestication genes provided evidence to challenge the above hypotheses (Sang and Ge, 2007; Sweeney and McCouch, 2007; Sweeney et al., 2007; Tan et al., 2008), which initiated some questions, such as why are most of the domestication genes identical in dispersed subgroups in various eco-systems? And how did the subgroups originate in distinct geographical regions? Genome-wide molecular markers and sequence data supplied more evidence about the phylogenetic relationship among Asian cultivated varieties, *O. rufipogon* and *Oryza nivara* accession, which provided strong clues to the origin of different subgroups of *O. sativa* when its geographical scope is expanding (Huang et al., 2012; Civán et al., 2015; Kim et al., 2016; Moner et al., 2018; Choi et al., 2020; Gutaker et al., 2020). The recent advances in rice genetics and genomics enable us to better understand the process of improving genetic diversity and environmental adaptability of Asian cultivated rice through hybridization-introgression during its evolution process. In this study, we reviewed the stories of *O. sativa* about its domestication, distribution, introgression, variation, and eco-climatic-economic adaptation, especially focusing on how the crop became better and better to meet the challenges of changing environments through continuous interspecific hybridization-introgression with its wild relatives, and genetically exchange and recombine to improve its genetic diversity and environmental adaptation ability, thus ultimately formatted the most important crop containing several subgroups to adapt various cultivation environments, to meet the increasing food requirement of expanding human population.

## Environmental Adaptation and Natural Hybridization Led to the Origin of Different Subgroups in *Oryza sativa*

### Asian Cultivated Rice Is a Wide Distribution Species With High Diversification

Compared to the hunter-gatherer system, cultivating domesticated crops can feed the more human population. Ten thousand years ago, human societies began to transition from hunting-gathering to agriculture. Both archeological and archeobotanical evidence show that the Asian cultivated rice domestication began in the Yangtze Valley in China approximately 8,000–8,500 years ago (Higham and Lu, 1998; Fuller et al., 2009). Rice is widely cultivated from 55*^o^*N in China to 36*^o^*S in Chile, and grown under different conditions such as irrigated, rainfed lowland, rainfed upland, and flood-prone ecosystems (Khush, 1997). It was estimated that more than 4,120,000 rice cultivars and germplasm accessions have been recognized worldwide (Song et al., 2021). As one of the earliest domesticated cereal crops in the world, with its spread of cultivation, rice appears more and more abundant in morphological and genetic diversity to adapt to the various diverse and complex cultivation environments.

Cultivars of *O. sativa* were first classified into two major types, namely *indica* and *japonica*, according to morphological and serological characters as well as inter-varietal hybrid fertility (Kato et al., 1928). Based on morphological traits, *O. sativa* was further classified into three types, A, B, and C (Matsuo, 1952), named *indica*, *javanica*, and *japonica* (Morinaga, 1954). Oka (1958) subsequently referred to the above three types as *indica*, *tropical japonica*, and *temperate japonica* groups. Using 15 polymorphic loci coding for eight enzymes, 1688 traditional rice varieties from Asia were classified into six varietal groups (Glaszmann, 1987), and it roughly represents different ecological types and geographical distribution. Two hundred and thirty-four accessions of rice were classified into five groups, corresponding to *indica, aus, aromatic, temperate japonica*, and *tropical japonica* by 169 nuclear SSR markers and two chloroplast loci (Garris et al., 2005). The classification of five subgroups was confirmed and accepted by subsequent studies that resulted from analysis of nuclear and cytoplast DNA markers, as well as genomic datum (McNally et al., 2009; Wang et al., 2018; Chen et al., 2019). However, some authors used *aromatic* to refer scented rice from *indica*, *temperate japonica*, *tropical japonica*, *aus*, and *aromatic* (Bourgis et al., 2008), while some authors used *aromatic* to refer a special subgroup from Southern Asia (Garris et al., 2005; Kovach et al., 2007). To avoid confusion, we used *basmatic* to refer this subgroup in this paper.

### *Temperate* and Tropical *japonica* Were the Results of Environmental Adaptation and Selection

The *japonica* is the first subgroup of fully domesticated Asian cultivated rice (Huang et al., 2012; Choi et al., 2017), and it was largely confined to China in the early cultivation stage (Khush, 1997). The *temperate japonica* was the first major population separated from tropical landraces (Gutaker et al., 2020). Rice diversified into *temperate* and *tropical japonica* rice during a global cooling event about 4,200 years ago (d’Alpoim Guedes et al., 2015). Standing variation of cold tolerance genes underwent stepwise selection to facilitate cold adaptation to expand rice cultivation from high altitude to high latitude regions (Li et al., 2021). The Hap*^tej^* of *COLDF* (*CHILLING-TOLERANCE DIVERGENCE F-box*) is responsible for enhanced cold tolerance in the *temperate japonica* cultivars (Zheng et al., 2022). *Temperate japonica* is the result of adaptation to cooling climatic conditions, and dispersed in North and Northeastern Asia, while *japonica* rice migrated to Southeast Asia, where it rapidly diversified as *tropical japonica* nearly 2,500 years ago (Gutaker et al., 2020).

### The *indica*, *aus*, and *basmatic* Varieties Were Produced by Hybridization and Introgression

Some domesticated traits, such as erect growth, loss of grain shattering, shortened awns or awnless, and compact panicles, are essential for the transition from wild rice to the domesticated crop, and these key transitions occurred in the earliest steps of rice domestication. Grain dormancy, grain color, grain size and number, and environmental adaptability were selected during the post-domestication stage (Xu and Sun, 2021). Dissection of allelic variants of essential domestication genes can reveal strong evidence about the origins of several subgroups in *O. sativa*. Most of the widely disseminated domestication alleles appear to have originated in *japonica* or a *japonica*-like ancestor and were subsequently introgressed into the *indica* group (Kovach et al., 2007). *prog1* is a single-origin allele and was selected during Asian rice domestication (Jin et al., 2008; Tan et al., 2008). A 110-kb deletion closely linked to *PROG1*, referred to as *rapd*, also contributed to the transition from prostrate to erect growth in Asian cultivated rice; this 110-kb deletion also represents a single evolutionary event, as all tested Asian cultivated rice carries it (Wu et al., 2018). The identical allele of *prog1* and *rapd* across *O. sativa* strongly suggests that introgression occurred after the spread of cultivated rice. The *sh4* allele originated in the early stages of Asian rice domestication (Li et al., 2006; Lin et al., 2007; Zhang et al., 2009) and introgressed into all the progenies as the crop expanded. A similar example occurred in rice pericarp color. The main mutation *rc* allele originated in the *japonica* and moved into the *indica*, *aus*, and *basmatic*. A fragment of no more than 1 Mb of *japonica* DNA with the *rc* allele hitchhiked into most *indica* varieties (Sweeney et al., 2007).

**FIGURE 1:**
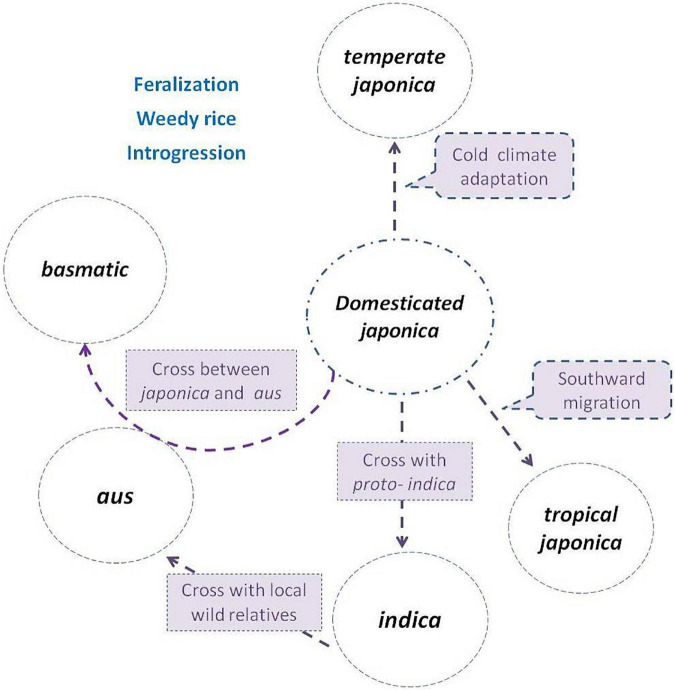
Subgroup differentiation of *Oryza sativa* in the evolution process. The *japonica* rice (circle in the center) was domesticated first from *Oryza rufipogon*, then divided northward as *temperate japonica*, and south ward as *tropical japonica*. The *indica* was descent from hybridization between *japonica* and local wild populations or *proto-indica.* The *aus* was derived from the hybridization between *indica* and local wild populations, while the *basmatic* was derived from hybridization between *japonica* and *aus*. Weedy rice strains de-domesticated from and coexisted with cultivated subgroups, and frequently crossed with wild populations (if present) or landraces during their evolution process.

Evidence combining agricultural production, archeology, genetics, nuclear, and cytoplasm genome datum supplied a strong indication that subgroups *indica*, *aus*, and *basmatic* were descended from distinct lineages (Civán et al., 2015; Carpentier et al., 2019; He et al., 2021; Zhang et al., 2021), accompanying hybridization and introgression events, and both ancient and more recent gene flow continues to dilute the once-distinctive gene pools (Kim et al., 2016; Wang et al., 2017). Thus, the course of origin in *indica*, *aus*, and *basmatic* has been gradually clear (Figure 1).

The *indica* rice is with the most abundant genetic diversity, widely geographically distributed, and adapted in tropical and subtropical regions. *O. rufipogon* and *O. nivara* are native to India and well-distributed there today. The archeological data for the Ganges River basin reveals that sedentary agricultural villages were established after 2500 B.C. (Fuller, 2006; Fuller et al., 2010); thus, *proto-indica* rice was of unclear domestication status in South Asia before the domesticated *japonica* was introduced into India. Domesticated *japonica* rice dispersed from China, south then west ward, via Myanmar, Assam, Bangladesh to India Plain (Kovach et al., 2007; Vaughan et al., 2008), or from China, northwest ward, via Hexi Corridor through the Silk Road trade, to Pakistan, to Northwest India, then the Indian Plain (Fuller et al., 2010; Fuller, 2011b; Silva et al., 2018). Once *japonica* was present alongside local unimproved *proto-indica* or wild populations, hybridization, and back-crossing could rapidly induce the origin of *indica* subgroup by selection regimes of farmers. The complete domestication of *indica* was only when the arriving of domesticated *japonica* from China and hybridized with local wild relatives or *proto-indica* about 4,000-5,000 years ago (Fuller, 2011a; Gross and Zhao, 2014). As a result, the *indica* subgroup contained the most genetic diversity, harboring almost all (seven of the eight) chloroplast haplotypes, and encompassing all the chloroplast diversity found in the *temperate* and *tropical japonica* (Garris et al., 2005).

The *aus* rice is mainly distributed across South Asia, mainly concentrated in the Indian subcontinent. The *aus* group is grown under rain-fed upland and lowland conditions, and some are adapted to the irrigated or deepwater cultivated system. It likely originated from the hybridization between *indica* and a local wild population, with the identical recessive bacterial blight resistant gene, *xa-5*. Some *aus* varieties still remain some characters of wild relatives of rice, such as spreading tillers and easy thresh (Khush et al., 2003), and are under incomplete domestication selections (Zhao et al., 2018). Molecular and genomic studies suggested that the *aus* rice was originated from annual *Oryza nivara* (Kim et al., 2016), with distinct lineages of both nuclear and cytoplasm inheritance (Civán et al., 2015; Choi et al., 2017; He et al., 2021) and is relatively close to *indica* (Garris et al., 2005; Moner et al., 2018, 2020; He et al., 2021).

The *basmatic* rice is a small subgroup of *O. sativa*, identified as group V by Glaszmann (1987), including “Basmati” and “Sadri” varieties. The pleasure fragrance of rice genetically results from a recessive allele of *badh2.1* on chromosome 8 (Bourgis et al., 2008). Sequence analysis around *BADH2* indicated that they contain *badh2.1* allele originating from *japonica* (Kovach et al., 2009). The basmati rice, a typical representative of the *basmatic* group, is characterized as extra-long slender grain, elongation on cooking, soft and fluffy texture of cooked rice, and a pleasant aroma. The earliest reference to *basmatic* rice in India can be traced to the documents before 2,400 years ago. The *basmatic* is now a geographical indication, grown in the north-western foothills of the Himalayas in the Indian subcontinent, and is attributed to a unique combination of genetics, soil, water, climate, and cultural practices (Siddiq et al., 2012). Both the nuclear and chloroplast data demonstrate a close relationship to *japonica* (Garris et al., 2005). The *japonica* rice contributed the highest amount of genetic material to circum-*basmatic*, and strong evidence of admixture between the circum-*basmati*c and circum-*aus* groups was detected (Choi et al., 2020). A unique pericarp color haploid, *rc-s*, is shared only in *aus* and *basmatic* subgroups (Sweeney et al., 2007). The *basmatic* rice is a result of a hybridization between *japonica* and *aus* (Civán et al., 2015; Kishor et al., 2020).

Therefore, although a genetic bottleneck occurred in the early domestication stage, subsequent hybridization-introgression enabled *O. sativa* to enlarge its genetic diversity and differentiated into several subgroups to adapt to various cultivation environments. It is believed that Asian cultivated rice has the evolution progress of “One domestication and multiple origins” or “One origin and multiple interspecific introgressions.” The *indica*, *japonica*, *aus*, and *basmatic* were parallel in origin, but gene introgressions among each other happened (He et al., 2021). The main domesticated genes were from *japonica* rice. Selective sweeps with reduced diversity were widespread in both *indica* and, in particular, *tropical japonica* (Molina et al., 2011). That is, there was only one domestication event in *O. sativa* (Gross and Zhao, 2014).

### Weed Rice Is a Cospecific Form of Cultivar Rice, Resulted From Hybridization, Feralization, and Introgression, Adapting to the Improving Cultivation Environments

Gene flow from a crop plant to its wild progenitor is particularly effective when it helps the latter to mimic the crop (Ladizinskv, 1998). When wild relatives are growing near the farmers’ field, they can cross with cultivars and form weedy types, which can be a serious problem to the farmers, particularly when the rice is broadcast sown, such as in deepwater rice fields in Asia river deltas (Vaughan and Sitch, 1991). Genes from cultivated rice can be easily incorporated into the gene pool of weedy rice through recurrent crop-to-weed gene flow and introgression. This progress may promote the persistent and better adaptation of weedy rice to the human-influenced habitats (Harlan, 1965). Typical specimens of *O. rufipogon* and *O. nivara* are rarely obtainable today even in their adapted habitats because of continuous hybridization among the wild, weed, and cultivated races (Chang, 1976). As wild species have been a serious weed in rice fields in India, the purple-leaved variety was introduced to allow the farmers to weed out the spontaneous green-leaved wild form in the field. However, no more than 20 years later, the purple-leaf gene had been introduced to the weedy population to such an extent that weeding according to leaf color was no longer a safe practice for eliminating wild species (Dave, 1943). Weedy rice now occurs worldwide and is prevalent in mechanized, direct-seeded farming systems. Genetic surveys have revealed multiple independent origins of weedy rice around the world. When wild *O. rufipogon* or *O. nivara* were absent, de-domestication from rice varieties or landrace plays a major role in the formation of weedy rice in northern temperate rice-growing regions. Interestingly, in the United States, almost all weedy strains de-domesticated from *aus*, *indica*, and *japonica* which originated and grew outside of the United States. When *O. rufipogon* or *O. nivara* was present in South Asia, and Southeast Asia, weedy rice strains were morphologically diverse, with characteristics ranging from resembling cultivated rice to similarity to wild rice, and showed evidence of recent hybridization between domesticated (or de-domesticated) and wild populations (Li and Olsen, 2020; Vigueira et al., 2020).

## Artificial Interspecific Hybridization and Introgression Led to Several Breakthroughs in Rice Breeding

### Introgression of Sterile Cytoplasm From *Oryza rufipogon* to *Oryza sativa* Is the Start Point for the Three-Line Hybrid Rice Revolution

Chinese breeders began researching the utilization of rice heterosis in 1961. However, male-sterile plants found in cultivated rice are difficult to be maintained. In the Winter of 1970, a male-sterile plant was identified in *O. rufipogon* in Hainan Province, China. The cytoplasm of this male-sterile (CMS) *O. rufipogon* plant was introduced into *O. sativa* nuclear background, and successfully bred a series of wild abortive male sterility lines, which led to the famous “green revolution” of hybrid rice. By 2019, more than 7,000 hybrid rice cultivars had been released in China, with a total planting area of about 6 × 10^8^ ha^2^ and an increased rice grain of nearly 9 × 10^8^ t, which had fed an additional 2.3 billion people. Chinese hybrid rice has been successfully tested or developed in more than 60 countries with planting areas exceeding 6 × 10^6^ ha^2^ (He et al., 2020).

### New Rice for Africa Derived From Interspecific Hybridization Between *Oryza sativa* and *Oryza glaberrima*, Adapting Low Input Cultivation Conditions in West Africa

African cultivated rice *O. glaberrima* has been cultivated in West Africa for approximately 3,000 years (Linares, 2002). African rice has some undesirable features, such as seed shattering, brittle grain, lower yield potential, lodging, long seed dormancy, and being replaced by the Asian species, which was introduced into the continent by the Portuguese as early as the mid-sixteenth century (Jones et al., 1997; van Andel et al., 2016). The African rice has been described as the least diverse crop species ever documented (Nabholz et al., 2014). Despite losing substantial genetic diversity during domestication, it still retained important traits such as weed competitiveness, tolerance to drought, resistance to African gall midge, rice mottle virus, stem borer, and nematodes, as well as adaptation to acidic and low phosphorus soils among others (Wambugu et al., 2013). Although modern Asian cultivars performed better than farmers’ traditional *O. glaberrima* varieties under relatively higher input conditions, they performed poorly when cultivated under the low input systems which dominate extensive upland farming in West Africa, due to the characteristics of modern Asian cultivars, such as poor competition with weeds and limited resistance to many of the stresses that affect upland rice in the region (Jones et al., 1997). Interspecific hybridization between *O. sativa* and *O. glaberrima*, subsequently backcrossing with *O. sativa*, produced progenies combining advantages of both species (high yield potential, weed-suppressing, and adaptation to low input conditions) and a new rice type to adapt West Africa upland condition was developed, denoted as NERICA (new rice for Africa), and this led to the naming of many rice cultivars for both rainfed upland and lowland irrigated system of West Africa (Jones et al., 1997; Nayar, 2010).

### Miscellaneous New Genes Utilization and Breeding Achievements by Interspecific Introgression

During the 1970s, the grassy stunt virus is prevalent in several countries. Severe yield losses occurred under epidemic conditions. Of the 6,723 cultivated rice accessions and several wild species screened for resistance, only one *O. nivara* accession Acc. 101508 was found to be resistant (Ling et al., 1970). A dominant resistant gene from *O. nivara* was denoted as *Gs* (Khush and Ling, 1974). Resistant varieties with *Gs*, such as IR28, IR29, IR30, IR32, IR34, IR36, and subsequent varieties were released in the prevalent regions, and grassy stunt infected plants are now rarely observed in farmers’ fields (Khush and Brar, 2017).

Bacterial blight disease (BB) is one of the most serious diseases of rice in Asia. A dominant gene *Xa21* for resistance to BB was transferred from *O. longistaminata* and was widely used in many varieties in the Philippines, India, China, and other countries.

Recently, the progress of mining favorable genes from wild relatives involves more traits and genes/alleles. Great progress has been made in identifying genes/alleles resistant to biotic stresses. For blast resistance, *Pid3* from *O. rufipogon*, *Pi54rh* from *O. rhizomatis*, *Pi54* from *O. officinalis*, *Pi57*(t) from *O. longistiminata*, *Pi68*(t) from *O. glumaepatula*, and *Pi 69*(t) from *O. glaberrima* were identified (Das et al., 2012; Lv et al., 2013; Devanna et al., 2014; Xu et al., 2015; Devi et al., 2020; Dong et al., 2020). For bacterial blight, one novel resistant gene *xa-45*(t) from *O. glaberrima* was identified (Neelam et al., 2019). For sheath blight resistance, one major QTL *qShB9-2* was mined from *O. meridionalis* (Eizenga et al., 2013). For brown plant hopper (BPH) resistance, *qBph3* and *qBph4* from *O. officinalis*, *Bph34* from *O. nivara*, and *Bph27, Bph35*, and *Bph36* from *O. rufipogon* were identified (Huang et al., 2013; Hu et al., 2015; Kumar et al., 2018; Li et al., 2019; Zhang et al., 2020).

Some elite cultivars, such as Yundao1 [(IRGC102203 (*O. glaberrima*)/Boro5//Dianxi1///Hongza135], Yunlu142 [Yundao1/Acc.104613(*O. barthii*)//Yundao1/3/Yundao1] were bred via interspecific hybridization and released at Yunnan Academy of Agricultural Sciences (YAAS), Yunnan province, China.

## Interspecific Introgression Line Is Not Only a Resource for Favor Allele Mining but Also an Effective Way to Analyze the Genetic Basis of Homogeneous Genes in Cultivated Rice

The genetic difference between *O. sativa* and wild species has been observed in many studies. The hybrid progenies between cultivated and wild species usually showed abundant phenotypical and genotypical variation in various traits in segregated populations. The developing rice genomics has greatly contributed to the inherent nature of rice genetics and variation. However, focusing on a single trait or gene is convenient to elucidate its genetic nature by excluding disturbs of other genetic variances. Development of introgression libraries containing a single or few segments from wild species of rice into the cultivated background is the benefit to evaluate and dissect the genetic effect and breeding value for each genetic unit one by one. Series introgression libraries have been developed. Some libraries are single substitute lines (CSSLs) or backcross inbred lines (BILs) from a single donor, including *O. glaberrima* (Doi et al., 1997; Shim et al., 2010; Yamagata et al., 2019), *O. rufipogon* (Tian et al., 2005; Furuta et al., 2014; Qin et al., 2018; Ma et al., 2019; Yamagata et al., 2019), *O. longistaminata* (Ramos et al., 2016), *O. nivara* (Furuta et al., 2016; Yamagata et al., 2019), *O. meridionalis* (He et al., 2017), *O. bathii* (Bessho-Uehara et al., 2017; Zhao et al., 2019), and *O. glumaepatula* (Zhao et al., 2019). Compared with the rich genetic diversity of wild relatives of Asian cultivated rice, the introgression library with a single donor only represents a small part of its genetic diversity. Considering the great potential of genetic diversity in wild species, in recent studies, a multi-donor method was employed. In one study, 70 accessions of six AA genome species, *O. glaberrima*, *O. barthii*, *O. nivara*, *O. rufipogon*, *O. longistaminata*, and *O. glumaepatula*, were crossed with two elite cultivars of *O. sativa* L., then 1780 backcross inbred lines (BILs) were generated, and 15 BILs showed >10% yield superiority over the recurrent parents, with yield-related traits (Bhatia et al., 2017). A single-segment substitution lines (SSSLs) library, which includes 2360 SSSLs derived from 43 donors of seven species of rice AA genome in the Huajingxian 74 (HJX74) genetic background, was widely used to detect QTLs for traits of agronomic importance, clone genes of functional importance, and mine alleles of functional variants (Zhang, 2021). Recently, researchers developed 26,763 advanced backcross families using 309 donors of *O. glaberrima*, *O. barthii*, *O. nivara*, *O. rufipogon*, *O. longistaminata*, *O. glumaepatula*, *O. meridionalis*, and *O. sativa* (upland rice varieties). Based on phenotype selection, 6,732 introgression lines (ILs) with agronomic and morphological variation were raised. Using this library, 22 alleles responsible for grain shape were newly found (Zhang et al., 2022).

Steady agronomic and genetic interventions are essential for sustaining productivity in intensive rice cropping. Further progress in rice genetic improvement lies not only in the identification of more favored genes/alleles but also in a deep understanding of the genetic pathways of rice growth and development, yield formation, environmental response, and so on. However, as a result of extreme domestication, many excellent traits/genes in cultivated rice have been highly homogeneous, and the differences among varieties are difficult to be found, which is not conducive to the in-depth dissection of these important traits and their genetic networks. Compared with cultivated rice, wild relatives of the Asian cultivated rice have not been artificially domesticated and selected, they retain more genetic diversity, and the significant genetic difference between wild relatives and the Asian cultivated rice is an effective tool to analyze these genetic networks. Therefore, on one hand, the abundant genetic diversity is the elite resource for favor allele mining; on the other hand, as a result of long-term domestication and selection, many genes in cultivated rice are difficult to be characterized due to the lack of allelic differences. Exploring the huge genetic difference between cultivated rice and wild relatives is an effective way to analyze the genetic basis of these genes. The genetic dissection of *PROG1* gene using the genetic difference between *O. sativa* and *O. rufipogon* is a good example (Jin et al., 2008; Tan et al., 2008). Thus, interspecific hybridization-introgression is not only useful for Asian cultivated rice to be high diversity and resilience to the changing condition but also useful for new gene discovery and genomics development, which makes rice to be a model crop for genomics development.

## Prospect

**FIGURE 2:**
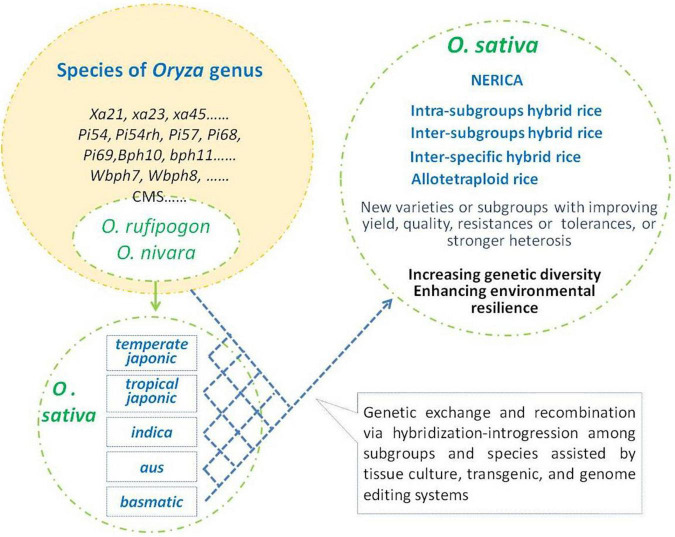
Artificial hybridization-introgression for genetic improvement of *Oryza sativa. O. sativa* group was descent from *Oryza rufipogon* and *Oryza nivara.* Species of *Oryza* genus including *O. rufipogon* and *O. nivara* are rich in genetic diversity and favorable traits/alleles. Some elite genes/alleles were transferred into Asian cultivated rice and gained several breakthroughs in rice breeding. Further hybridization-introgression among interspecies and inter-subgroups will provide more chances to enlarge genetic diversity and gain breeding achievements in *O. sativa*.

Thousands of years of rice evolution have made great progress in increasing yield, meeting human eating preferences, and adapting to a variety of cultivation environments. In this process, interspecific hybridization-introgression played important impact on the improvement of adapting to different environments and ensuring the genetic diversity of rice (Figure 1). During this progress, more and more favor alleles or mutations have been accumulated in cultivars to improve the performance of the Asian cultivated rice. The progress of breeding programs, especially breakthrough progress, lies in further more favorable gene utilization. However, most of the naturally occurring variants in rice are of low frequency (Zhao et al., 2018). Thus, interspecific hybridization will continuously play an important role in the introgression of favor genes/alleles and increase its genetic diversity in the future practices of genetic improvement in rice. With the intensification of modern cropping systems, most of the wild relatives of *O. sativa* have lost their natural habitats, and interspecific hybridization-introgression in genetic diversity improvement of Asian cultivated rice would mainly depend on artificial implementation (Figure 2).

Artificial interspecific hybridization has made great progress in the genetic improvement of cultivated rice. However, due to many difficulties in distant interspecific hybridization and introgression, the progress in genetic improvement of Asian cultivated rice by interspecific hybridization is not comprehensive. First, due to strict reproductive barriers, compared with non-AA genome species, wild relatives with the same AA genome as the Asian cultivated rice are easier to obtain interspecific hybridization offspring with Asian cultivated rice. Therefore, more favorable genes/alleles have been found from wild relatives with the AA genome, while few favorable genes/alleles have been found from the non-AA genome. Second, because wild species grew in the natural environment for a long time, resistance/tolerance to many bio- and abio-stresses is a necessary condition for their survival. It is easier for us to find resistant/tolerant genes from them. The yield-related traits of rice are mostly quantitative traits, which are easy to be affected by environment and genetic background. The expression of these yield traits was unstable among individuals/generations. Although there were many reports on rice yield-related genes/QTLs (Gaikwad et al., 2021), the progress is limited. Third, many favorable genes from wild relatives have been discovered and cloned, but few of them are used in breeding practice. Further efforts should focus on several respects in the future.

### Understanding and Overcoming Reproductive Barriers Between *Oryza sativa* and Wild Relatives, Including AA and Non-AA Genome Wild Relatives

For non-AA genome wild relatives, overcoming the reproductive barriers between them and the Asian cultivated rice is conducive to exploring a wider range of genetic variation. For AA genome relatives, in addition to exploring their genetic variation, the potential heterosis between the Asian cultivated rice and its relatives of the AA genome can be expected. Hybrids between varieties within subspecies of *indica* or *japonica* have shown great heterosis in the past half-century, and hybrids between subspecies or subgroups can gain more heterosis than the former. However, the hybrid sterility frequently occurred and hampered the utilization of the distant heterosis. Recent research demonstrated an example to overcome the inter-subspecies hybrid sterility (Guo et al., 2016), which greatly enhanced the confidence in the utilization of inter-subspecific heterosis between *indica* and *japonica*. The interspecific genetic difference between *O. sativa* and its relatives (including another cultivated species *O. glaberrima*) is greater than inter- and intra-subspecies, which show stronger hybrid vigor (Hu et al., 2002; Sun et al., 2020). Previous studies demonstrated that genetic knowledge about interspecific hybrid sterility can help us overcome this obstacle between *O. sativa* and its relatives (Deng et al., 2010; Xie et al., 2017; Yu et al., 2018). Future research needs to take efforts for the comprehensive identification of interspecific hybrid sterility genes between Asian cultivated rice and its relatives with potential heterosis, analyze their genetic mechanism, so as to overcome the obstacles of hybrid sterility and promote the utilization of interspecific vigor through the exploration of natural neutral alleles, and the development of bridge parents and gene editing of hybrid sterility genes.

### New Techniques Help Wider Interspecific Introgression

Some wild species have favor characteristics, such as salinity tolerance of *O. rufipogon*, high biomass production of CCDD genome species, *O. latifolia, O. alta*, and *O. grandiglumis* (Khush and Brar, 2017). However, several pre- or post-zygotic barriers, such as hybrid sterility, genomic disharmony, nuclear instability, hybrid breakdown, reversion to parental types, limited recombination, presence of deleterious genes, and linkage drag, hinder the production of remote interspecific hybrids and the transfer of genes into crops through interspecific hybridization (Brar, 2004). The new technologies help to introduce favorable genes from distant wild species. In crop breeding, transgenic technology is an effective system to introduce favorable genes from distant species. Thanks to the development of efficient tissue culture, transformation, genome editing and high-quality genome assembly technologies, it is possible that artificial de novo domestication of a wild plant in a few generations. Recently, artificial *de novo* domestication of a wild plant gained great progress, including in wild tomato (Li et al., 2018), orphan crop *Physalis peruviana* (Lemmon et al., 2018), and wild relative *O. alta* of rice (Yu et al., 2021). But, integration of new tools and interspecific hybridization can be mainstream to improve the Asian cultivated rice further in the future.

### In Addition to Interspecific Hybridization, Inter-Subgroups Hybridization Can Make the Asian Cultivated Rice More Adaptable

As the Asian cultivated rice differentiated into subgroups in various environments to meet multifarious cultivation conditions and eating preferences, many favorable traits and genes/alleles were raised in distinct populations. Introducing favorable alleles specific to one subgroup (such as quality, biotic- and abiotic-resistance/tolerance, and growth vigor) into other subgroups can enrich and improve their diversity in these traits. For instance, *basmatic* rice is usually with excellent rice qualities, its genetic elements of favor allele can be used to improve the quality traits of other subgroups. The *temperate japonica* rice at high altitude or high latitude usually has strong cold tolerance, and its genetic components of cold tolerance can also improve the adaptability of *indica* rice to cold climates. Similarly, the higher nitrogen-use efficiency (NUE) in *aus* and *basmatic* rice can also improve this trait of *japonica* and *indica* rice (Liu et al., 2021), so as to reduce the application of nitrogen fertilizer and then reduce input and environmental pollution. The premise of achieving the above purpose is to clarify the genetic basis controlling these traits.

Inter-subgroup hybrids can be another field to utilize heterosis in rice. Heterotic loci existed in wild progenitors of cultivated rice, and cultivated rice was divergently selected among rice subgroups. Divergent selection among the subgroups induced various heterotic alleles in different subgroups. Many heterotic loci in the current commercial hybrids were shaped by genome introgressions from different subgroups (Lin et al., 2020). Thus, introgression among subgroups could enhance heterosis in hybrid rice.

### Developing a Series of Introgression Lines With Favor Genes/Alleles From Wild Relatives in Various Subgroups Genetic Background Will Contribute to the Breeding Utilization of These Favorable Genes in Various Ecological Subgroups of Rice

In the past decades, many genes/alleles from wild relative were identified and even cloned, and the corresponding introgression lines (ILs) or near-isogenic lines (NILs) were developed in a specific genetic background. A specific genetic background has the adaptability to a given ecological environment; although distant hybridization can increase genetic diversity, when these ILs or NILs were crossed with parents of distinct genetic backgrounds to develop breeding populations, from the perspective of breeders, the offspring of distant hybridization usually perform poorly under a specific ecological condition. This may be the main reason for the limited utilization of these alleles in rice breeding projects. Yield-related traits usually are quantitative traits, and are often affected by both genetic background and environment, improving them in breeding programs needs to deal with more challenges. Therefore, developing a series of ILs or NILs with these favor gene/alleles in various specific genetic backgrounds will be conductive to reevaluate their genetic effect and breeding value in various genetic backgrounds and environments and to develop adaptive breeding populations.

## Conclusion

Asian cultivated rice originated from *O. rufipogon* and evolved thousands of years. During the domestication and evolution progress, when it gained favor mutations or was introduced into changing environments, only a few numbers of individuals could be retained and subsequently expanded, then the genetic bottlenecks occurred and genetic diversity decreased. Fully and long domesticated subgroup *japonica* showed a higher degree of differentiation and lower genetic diversity than *indica* (Zhu et al., 2006). Interspecific hybridization between domesticated rice and its wild relatives supplied chances to increase its genetic diversity, expanded and evolved in various subgroups, and adapted to more and more changing environments. Natural hybridization and introgression led to various subgroups of *O. sativa*, which adapted to changing cultivated environments, and produced feral weedy rice coexisting and competing with cultivars under cultivation management. Artificial interspecific hybridization and introgression gained several breakthroughs in rice breeding, such as hybrid rice, NERICA, and biotic stress resistance genes, which are contributed to the stable increase of rice production to meet the expanding human population. As interspecific hybridization-introgression have played important roles in improving genetic diversity and adaptation of *O. sativa*, more efforts need to make to exploit its virtues. Some key issues, such as reproductive barriers especially hybrid sterility should be investigated further, which are conducive to gene exchange between cultivated rice and its relatives, and even is beneficial to exploiting interspecific hybrid vigor. New techniques, such as efficient tissue culture, transgenics, genome editing system, and high-quality genome assembly, will help wide distant interspecific introgression and make the crop better. Compared with the huge genetic diversity of wild relatives, the construction of introgression lines in a wider range with more donors is needed, which benefits allele mining, understanding the genetic network of rice growth and development, yield formation, and environmental adaptation. Thus, developing a series of ILs or NILs with these favor gene/alleles in the various specific genetic background will be conductive to reevaluate their genetic effect and breeding value in various genetic backgrounds and environments and helpful to develop more usable breeding populations in various situations. In addition to interspecific hybridization, hybridization between inter-subgroups can make the Asian cultivated rice wider adaptable and stronger heterosis.

## Author Contributions

DT proposed the concept. JZ conceived and wrote the manuscript. YY, YL, QP, JL, YZ, XD, MW, and JW reviewed and edited the manuscript. All authors contributed to the article and approved the submitted version.

## Conflict of Interest

The authors declare that the research was conducted in the absence of any commercial or financial relationships that could be construed as a potential conflict of interest.

## Publisher’s Note

All claims expressed in this article are solely those of the authors and do not necessarily represent those of their affiliated organizations, or those of the publisher, the editors and the reviewers. Any product that may be evaluated in this article, or claim that may be made by its manufacturer, is not guaranteed or endorsed by the publisher.
